# Supplementary material for: Severe Scalp Psoriasis Microbiome Has Increased Biodiversity and Relative Abundance of Pseudomonas Compared to Mild Scalp Psoriasis
Source: J Clin Med. 2022 Nov 30;11(23):7133. doi: 10.3390/jcm11237133 (PMC9739726; doi:10.3390/jcm11237133)
Supplement: Supplementary file 1 [file jcm-11-07133-s001.zip › jcm-2020438-supplementary.pdf]

Supplemental Material

Supplementary Table S1. Descriptions of the Kyoto encyclopedia of genes and genomes (KEGG) pathways shown in Figure 5.

KEGG pathway	Description	P-value
K03704	cspA; cold shock protein (beta-ribbon, CspA family)	3×10^{-4}
K01999	livK; branched-chain amino acid transport system substrate-binding protein	5×10^{-4}
K00459	ncd2, npd; nitronate monooxygenase [EC:1.13.12.16]	7×10^{-4}
K01996	livF; branched-chain amino acid transport system ATP-binding protein	2×10^{-3}
K01998	livM; branched-chain amino acid transport system permease protein	2×10^{-3}
K07090	K07090; uncharacterized protein	2×10^{-3}
K01754	E4.3.1.19, ilvA, tdcB; threonine dehydratase [EC:4.3.1.19]	2×10^{-3}
K01995	livG; branched-chain amino acid transport system ATP-binding protein	2×10^{-3}
K03111	ssb; single-strand DNA-binding protein	3×10^{-3}
K00249	ACADM, acd; acyl-CoA dehydrogenase [EC:1.3.8.7]	3×10^{-3}
K00626	E2.3.1.9, atoB; acetyl-CoA C-acetyltransferase [EC:2.3.1.9]	4×10^{-3}
K02003	ABC.CD.A; putative ABC transport system ATP-binding protein	5×10^{-3}
K01997	livH; branched-chain amino acid transport system permease protein	6×10^{-3}
K00382	DLD, lpd, pdhD; dihydrolipoamide dehydrogenase [EC:1.8.1.4]	7×10^{-3}
K00666	K00666; fatty-acyl-CoA synthase [EC:6.2.1.-]	8×10^{-3}
K00257	mbtN, fadE14; acyl-ACP dehydrogenase [EC:1.3.99.-]	9×10^{-3}

KO, KEGG ortholog.

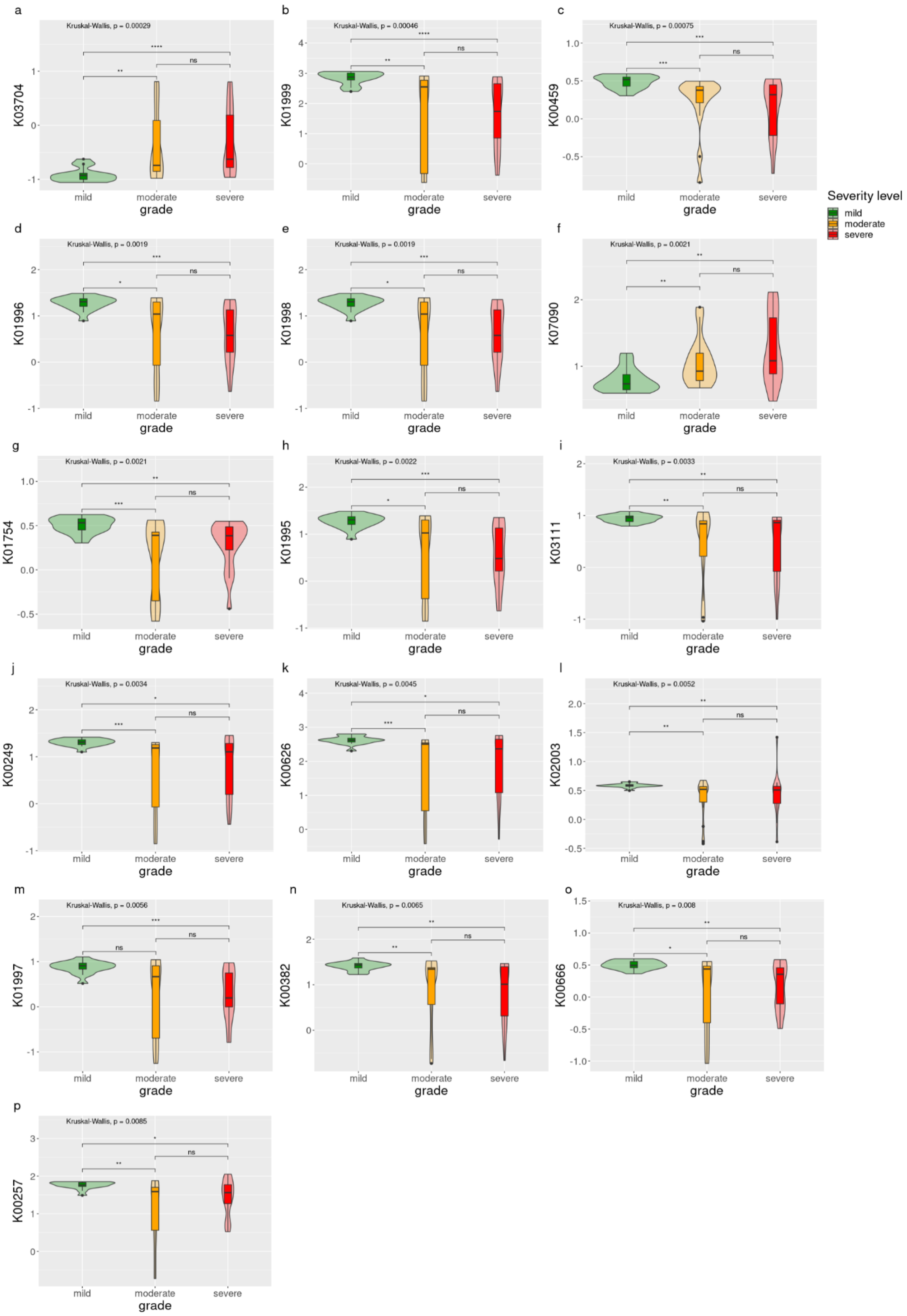

a
Kruskal-Wallis, p = 0.00029
K03704
b
Kruskal-Wallis, p = 0.00046
K01999
c
Kruskal-Wallis, p = 0.00075
K00459
Severity level
mild
moderate
severe
d
Kruskal-Wallis, p = 0.0019
K01996
e
Kruskal-Wallis, p = 0.0019
K01998
f
Kruskal-Wallis, p = 0.0021
K07090
g
Kruskal-Wallis, p = 0.0021
K01754
h
Kruskal-Wallis, p = 0.0022
K01995
i
Kruskal-Wallis, p = 0.0033
K03111
j
Kruskal-Wallis, p = 0.0034
K00249
k
Kruskal-Wallis, p = 0.0045
K00626
l
Kruskal-Wallis, p = 0.0052
K02003
m
Kruskal-Wallis, p = 0.0056
K01997
n
Kruskal-Wallis, p = 0.0065
K00382
o
Kruskal-Wallis, p = 0.008
K00666
p
Kruskal-Wallis, p = 0.0085
K00257
mild
moderate
severe
grade

Supplementary Figure S1. Violin plots of predicted activity of each KO pathway according to the severity of scalp psoriasis. Bacterial gene functions were predicted from 16S rRNA gene-based microbial compositions using the PICRUSt algorithm. Of 63 KO pathways tested, 16 pathways differed in abundance between mild, moderate and severe groups (a-p) ($p<0.01$). Green indicates mild group; yellow indicates moderate group; red indicates severe group. Full lists of KO pathways can be found in Supplementary Table S1.

**** $p <0.0001$, *** $p<0.001$, ** $p<0.01$, * $p<0.05$, ns $p>0.05$

KO pathways with KO, Kyoto encyclopedia of genes and genomes **(**KEGG**)** ortholog.
